# Daily testing of contacts of SARS-CoV-2 infected cases as an alternative to quarantine for key workers in Liverpool: A prospective cohort study

**DOI:** 10.1016/j.eclinm.2022.101519

**Published:** 2022-07-01

**Authors:** Lucy Marsden, David M. Hughes, Rhiannon Corcoran, Christopher P. Cheyne, Matt Ashton, Iain Buchan, Emer Coffey, Marta García-Fiñana

**Affiliations:** a Public Health Department, Liverpool City Council, Liverpool, Cunard Building, Water Street, Liverpool, L3 1DS; b Department of Health Data Science, Institute of Population Health, University of Liverpool, Liverpool, UK; c Department of Primary Care and Mental Health, Institute of Population Health, University of Liverpool; d Department of Public Health, Policy and Systems, Institute of Population Health, University of Liverpool, Liverpool, UK

**Keywords:** COVID-19, Key-workers, Lateral flow tests

## Abstract

**Background:**

Covid-19 test-to-release from quarantine policies affect many lives. The SMART Release pilot was the foundation of these policies and an element of the world's largest population cohort study of community-wide, SARS-CoV-2 rapid antigen testing. The objective of the study was to evaluate daily lateral flow testing (LFT) as an alternative to 10-14 days quarantine for key worker contacts of known Covid-19 (or SARS-CoV-2 infection) cases.

**Methods:**

Prospective cohort study incorporating quantitative and qualitative research methods to consider how serial LFT compares with PCR testing to detect SARS-CoV-2 infections and to understand experiences/compliance with testing and the viability of this quarantine harm-reduction strategy. Participants were residents of the Liverpool area who were key workers at participating fire, police, NHS and local government organisations in Liverpool, and who were identified as close contacts of cases between December 2020 and August 2021. Thematic qualitative analysis was used to evaluate stakeholder meetings.

**Findings:**

Compliance with the daily testing regime was good across the three main organisations in this study with 96·9%, 93·7% and 92·8% compliance for Merseyside Police, Merseyside Fire & Rescue Service and Alder Hey Children's Hospital respectively. Out of 1657 participants, 34 positive Covid-19 cases were identified and 3 undetected by the daily LFT regime. A total of 8291 workdays would have been lost to self-isolation but were prevented due to negative daily tests. Organisations reported that daily contact testing proved useful, flexible and well-tolerated initiative to sustain key worker services.

**Interpretation:**

Compliance with daily testing among key workers was high, helping sustain service continuity during periods of very high risk of staffing shortage. Services reported that the pilot was a “lifeline” and its successful delivery in Liverpool has been replicated elsewhere.

**Funding:**

This report is independent research commissioned by DHSC and part funded by DHSC and NIHR. Further funding was received from Liverpool City Council, the EPSRC and MRC.

**Research in context**

**Evidence before this study**

The performance of lateral flow tests (LFTs) is well documented, but the use of LFTs for daily testing to release contacts from quarantine, including compliance and the impact on services, is less well reported. The City of Liverpool was selected by the UK Government to pilot large scale community testing when the City had the highest prevalence of Covid-19 in England. Part of this pilot included SMART release, which assessed the daily use of LFTs as an alternative to quarantine in key workers who were close contacts of known Covid-19 cases.

**Added value of this study**

This study adds the first qualitative and quantitative analysis of a City-wide, established daily testing alternative to quarantine for contacts of SARS-CoV-2 infected cases. We report that most Covid-19 cases were detected and a total of 8291 workdays that would otherwise have been lost to quarantine were secured due to negative daily LFTs, safeguarding critical services from what could otherwise have been severe disruption. Services reported that this SMART Release pilot was a “lifeline” and its successful delivery in the City of Liverpool has demonstrated how such schemes can be put in place elsewhere.

**Implications of all the available evidence**

This study was the foundation of the UK Daily Contact Testing or ‘test-to-release’ policy and is nested in the world's largest population cohort study of community-wide, SARS-CoV-2 rapid antigen testing. This study adds to the evidence which led to the development of Covid-19 test-to-release policies that affect many lives.

Alt-text: Unlabelled box

## Introduction

The daily functioning of key organisations has, and continues to be, disrupted by the Severe Acute Respiratory Syndrome Coronavirus 2 (SARS-CoV-2) pandemic with key staffing diminished by the need to quarantine following contact with a positive case. In England, at the time of this pilot, the standard protocol for key workers who have been in contact with a confirmed case of SARS-CoV-2 indicated the need to quarantine for 10 days since exposure (prior to 14th December 2020, 14 days of quarantine were required in England).^1^ Self quarantine of contacts of known SARS-CoV-2 cases aimed to reduce transmission by isolating individuals promptly, in case they became infectious. A recent study reported that out of the contacts who agreed to be tested following a call from NHS Test & Trace, 16·3% returned positive tests.^2^ This leaves a large group of individuals who are required to quarantine, despite not being infected by their contact.

The diagnostic standard for identification of clinical cases of SARS-CoV-2 are laboratory-based reverse transcription polymerase chain reaction (PCR) tests,^3^ which are impractical for maintaining key services due to the logistics of lab-based testing and results typically taking 24–48 h to report. Rapid point-of-care tests such as the Innova SARS-CoV-2 Antigen Lateral Flow Test (LFT) are designed to quickly identify people who have higher viral loads and who are therefore most likely to be infectious.^4^ Lateral flow devices typically give results within 30 minutes and can be carried out anywhere, without a laboratory, offering the potential to quickly identify SARS-CoV-2 infections, quarantine, and break transmission chains. For these reasons, the test offers a potential solution to allow key workers to attend work and reduce avoidable quarantine with a negative LTF result.^5^ A large study with asymptomatic participants showed that LFT has a sensitivity of 40% relative to PCR, but that it can detect >90% of individuals with PCR Cycle Threshold <18·3 (corresponding to an approximate viral load of >10^6^ RNA copies/ml - thought to be the most infectious individuals).^4^ Comparison of LFT and PCR sensitivity sparked controversy when proposed as an alternative to quarantine,^6^ however, the debates seldom considered the time to get results and practical deployment – hence ‘useful sensitivity’. Since a single LFT may miss around 1 in 10 likely infectious individuals, repeat testing was been proposed as an alternative to quarantine for those identified as close contacts of known Covid-19 cases or asymptomatic SARS-CoV-2 infections.^7^^,^^8^ A number of studies utilising clinician led testing have shown that LFTs are likely to perform accurately during the acute stage of infection.^9^, ^10^, ^11^ A small number of studies have used surveys to assess the acceptability to the general public of daily testing schemes as an alternative to isolation.^12^^,^^13^

From November 6th 2020, Liverpool City Council, the NHS and the University of Liverpool partnered with the Department of Health and Social Care (DHSC) to pilot rapid antigen testing with LFT open to all people living or working in the City of Liverpool.^14^ Parts of this pilot investigated the real world accuracy of LFT vs PCR,^4^ factors influencing the uptake of LFT,^15^ and the use of LFT testing strategies linked to care home access,^16^ and in school aged children.^17^ The sub-study reported here aimed to evaluate daily LFT as an alternative to quarantine for key workers in the City. The SMART Release protocol employed serial daily LFTs up to day 7 post exposure, plus a research/evaluation PCR, usually taken on day 6 or 7. We first assessed compliance with the testing regime amongst key-workers who were contacts of known Covid-19 cases. In addition, we reviewed any benefits of reducing avoidable quarantine and maintaining essential services, the ability to detect cases of Covid-19 using a series of LFTs, and the concerns and experiences of stakeholders in relation to daily testing including any behavioural, usability, administrative and organisational factors that might affect the testing process, and its impact on Covid-19 management.

## Methods

We undertook a descriptive epidemiological analysis of Covid-19 testing and case data, alongside an exploratory thematic analysis with participating organisations. The study was undertaken between 4th December 2020 (the date of the first participant) and 16th August 2021, at which point national procedures came into force.

The study protocol and a description of the pilot can be viewed in the online supplement. The pilot consisted of serial daily LFTs for key worker contacts of confirmed SARS-CoV-2 cases from the day of identification. Contact was officially defined by the UK Government at the time of the pilot as anyone who had the following types of contact with someone who had tested positive for Covid-19: face-to-face contact including being coughed on or having a face-to-face conversation within one metre, anyone who had been within one metre for one minute or longer without face-to-face contact, or anyone who had been within 2 metres of someone for more than 15 minutes (either as a one-off contact, or added up together over one day). Organisations invited to be involved in the pilot were those offering services key to the City's functioning.

First implementation began with Merseyside Police in December 2020, and later that month with Merseyside Fire and Rescue Service (MFRS). Alder Hey Children's Hospital, Adult Social Care (primarily domiciliary care providers) and Liverpool City Council joined in February 2021.

For organisations to join the pilot, a small project team led by the local Public Health Team (Liverpool City Council) developed an implementation pack comprising key documents including briefing notes, a template protocol with guidance, a monitoring spreadsheet, and consent statements. These documents ensured rapid implementation was possible, with organisations equipped for briefing staff, inviting them to participate, and to collate the necessary data. A steering group was established to oversee and support the extension of the pilot.

A PCR test was carried out for all participants at the end of their testing regimen for the purpose of evaluating the safety of the daily LFT testing regime. This initially took place at a pillar 1 testing centre (Hunter Street, Liverpool) and from February 2021, participants were expected to book a PCR test via the gov.uk website which would be delivered to their home address. In each case, the participant performed the PCR test themselves, using the standard testing instructions provided in PCR test kits. PCR tests were sent to Lighthouse Laboratories for PCR testing, which uses Thermo Fisher quantitative PCR equipment in a standardised protocol, and their standard ThermoFisher TaqPath™ RT-qPCR SARS-CoV-2 assay.

Participation in the pilot was voluntary. To be eligible to take part in this test-to-release scheme the key worker was required to work or live in the Liverpool area and have been identified as a close contact of a person confirmed to be positive for SARS-CoV-2. Individuals were not eligible if there was a confirmed SARS-CoV-2 case in their household or bubble (individuals were allowed to form small closed groups for support with a limited number of contacts from a different household in special circumstances such as having an infant under 1), or if anyone in their household or bubble had symptoms of Covid-19. Those who were not able to commit to daily testing were advised that they must continue to quarantine in accordance with Government guidance & NHS Test & Trace guidance.

Staff were expected to report to their organisational SMART Release co-ordinator if they were a contact of a positive case. Participants had to commit to undertake serial daily testing commencing on the first day they were identified as a contact until day 7 following their last contact with the positive case. The Innova lateral flow device was pre-selected by NHS Test & Trace and the only device available to the SMART-release pilot team at the time the study was started. It was also the device most used by Liverpool's general population throughout the study period. Tests were taken, either at asymptomatic testing centres, or at designated testing spaces within Merseyside Police and Merseyside Fire & Rescue Service. Participant at Alder Hey Children's Hospital could also use home test LFT kits (provided by the Trust), as that was part of their already established testing protocol. Photographic evidence of their LFT test was expected to be sent to their organisation. All testing was self-administered but assisted in the sense that trained staff were present at each site. Participants swabbed their throat and anterior nares. If their daily rapid LFT returned a negative result they were released from quarantine for 24 h either to attend work or to carry out routine activity permitted in accordance with the localised rules (lockdown or tier restrictions were in force during different phases of the pilot). Any individual who tested positive with LFT was required to isolate and obtain a PCR as soon as possible to confirm. In February 2021 the pilot was extended to cover all staff of Merseyside Police and Merseyside Fire & Rescue Service workers from the wider Merseyside area.

Adherence to the LFT testing protocol was evaluated by comparing the number of tests taken by a key worker with the number they were expected to have taken. For key workers who were identified as a contact on the day the contact took place, if there was 100% adherence then each key worker would have performed 7 LFTs (on days 1-7) and 1 PCR test (on day 6/7). It was possible for individuals to join later following exposure if they were not identified as a close contact on the day the contact took place. In this case, the individual was expected to take daily LFTs for the remaining period of the 7 days following the original contact with a known Covid-19 case.

The scheme was run until the submission of the full Liverpool Covid-SMART Community Testing Pilot^14^ report. At this time, national daily contact testing (DCT) studies led by DHSC and Public Health England (PHE) were running and key worker organisations were able to be signposted to these as part of an exit strategy. In June 2021, the scheme came to an end for the organisations with smaller numbers of participants, including Liverpool Street Scene Limited and Domiciliary Care providers. For organisations where staff were deemed to be working in critical roles and where there were significant concerns for workforce capacity due to rising Covid-19 prevalence, the scheme continued until 16th August 2021 (Alder Hey Children's Hospital, Merseyside Police and Merseyside Fire & Rescue Service).

Organisations monitored the daily test results and outcome of the confirmatory PCR using the monitoring spreadsheet included within their implementation pack. This was anonymised by each organisation on a weekly basis and shared with the Project Team (LCC) who reviewed it for accuracy and to check there were no gaps in the data. Any missing data was queried with the organisation who checked their reporting and added any missing fields. The data was then shared with University of Liverpool who carried out an independent quantitative analysis.

Power calculations were conducted within the context of Bayesian analysis. Under the assumption that the proportion of participants with a positive PCR follows a Beta distribution, and that daily lateral flow testing (as proposed in the protocol) detects ≥90% of the cases, sample sizes between approximately 1,000 and 5,000 are needed to detect, with 95% probability, a statistically significant reduction in the proportion of PCR cases on Day 6-7 (compared to a reference scenario with no daily lateral flow testing). Given the observed changes in prevalence over time, calculations were conducted assuming a range of Covid-19 prevalence values, varying from 0.1% to 0.4%. For 0.2% prevalence, for instance, the sample size is approximately n=2,000.

In this pilot, anyone who was eligible and agreed to participate was included in the cohort. The sample size achieved (n=1657) was sufficient to assess compliance and benefits of daily lateral flow testing, and the plan was to maximise the numbers for a meaningful assessment of the ability of daily lateral flow testing to identify cases.

### Ethical approval

Daily testing of key workers with Innova lateral flow devices was commissioned and authorised by DHSC. The secondary analysis of anonymised data from this testing was considered service evaluation not research by DHSC, and as such did not require research ethics committee review (see http://www.hra-decisiontools.org.uk/research/docs/DefiningResearchTable_Oct2017-1.pdf). The University of Liverpool Local Research Ethics Committee review and approve the Covid-SMART pilot evaluation. All participants were asked for consent prior to joining the pilot scheme.

### Statistical methods

Basic demographic summaries for the individuals tested in each organisation are reported including the number of participants, age, ethnicity and sex (where available) for each of the three major organisations included in this study (Merseyside Police, Merseyside Fire & Rescue and Alder Hey Children's Hospital). We do not report the smaller organisations due to concerns about identifiability.

We report the total number of LFTs taken per individual and the total number of tests taken on each day following contact with known Covid-19 cases. We summarise graphically, using bar charts, the number of days between contact with a known case and identification as a contact by NHS Test & Trace.

The number of working days saved using daily LFT testing is calculated as the total number of negative LFT tests obtained (since an individual could work on those days). We report the compliance with the daily testing regime by reporting the ratio of the number of LFT tests received (total number among participants) divided by the expected total number of LFT tests. The expected number of tests per individual is 7 minus the day on which they were identified as a case, except when an individual withdrew during the scheme or tested positive, in which case we expected a daily LFT from the day after notification until the date of withdrawal/positive test.

Given the low prevalence of Covid-19 cases, and hence low number of positive cases, during much of the pilot period, the sample size was insufficient to provide precise assessment of the accuracy of the daily testing scheme regarding PCR as the reference standard. However, for illustrative purposes we classified an individual as LFT negative if all their LFT tests were negative and LFT positive if any of their LFT tests were positive. With this assumption, a calculation of the sensitivity of the testing regime was made relative to the PCR result. Confidence intervals were estimated using the Clopper-Pearson exact method.

Qualitative methods were used to understand the experiences of each organisation and to consider contextual factors affecting participation and adherence. The data for thematic analysis was collected from representatives of the involved organisations, as researchers attended the SMART Release Steering Board on the 5th and 12th March 2021. These meetings focused on gathering evidence on any benefits, compliance, concerns, and experiences of participating organisations and stakeholders. Organisations and sectors involved included Merseyside Police, Merseyside Fire & Rescue, Domiciliary Care providers, Liverpool Street Scene Limited, Alder Hey Children's Hospital NHS Trust, and Liverpool City Council. The first meeting focused on the organisations who had well established SMART Release schemes in place – The Police and Fire and Rescue services. The second meeting focused on the involvement of smaller organisations that were earlier on in the process or who had recruited fewer to SMART Release. The domiciliary care providers were the dominant group in the second meeting with Liverpool Street Scene Limited providing useful insights into adapting the processes to meet the needs of their workforce. Each lasted approximately one hour. These sessions were not recorded, as per the established protocol for previous meetings of the steering board. Instead, detailed notes of the discussion were taken by two members of the qualitative research team. These notes were coded and collated into over-arching themes informed by Framework Analysis methodology. The combined notes were subjected to thematic analysis.

### Role of the funding source

This report is independent research commissioned by DHSC and part funded by DHSC and NIHR. The Department of Health and Social Care supported this work as part of the Liverpool Covid-SMART pilot evaluation, however had no involvement in the collection, analysis and interpretation of data, or the decision to submit for publication. All authors had full access to the data in the study and accept responsibility to submit for publication. IB, as lead investigator for the DHSC Covid-SMART pilot is guarantor for the study and has accessed and verified the data.

## Results

**Figure 1:**
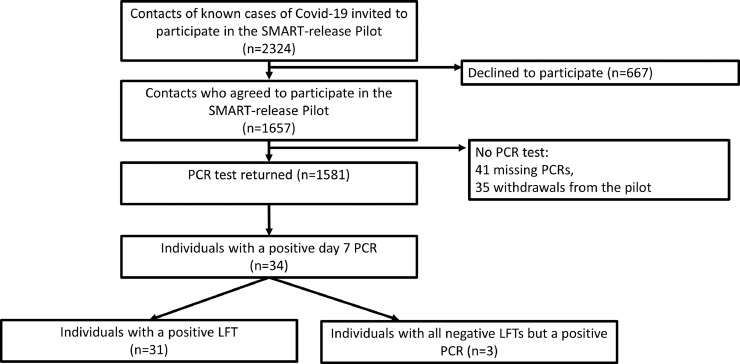
Inclusion Flowchart showing participants in the SMART-release pilot.

Between 4th December 2020 and 16th August 2021, a total of 2324 individuals were invited to participate in the pilot. Of these 1657 individuals enrolled on this key-worker-release scheme. 1581 individuals completed the day 7 PCR, and 34 of these PCR tests were positive. Of the 34 individuals identified as positive, 31 had a positive LFT during the daily testing regimen. The number of individuals recruited per organisation, and a summary of the number of tests per day since original exposure is shown in Table 1. A flowchart showing participation is shown in Figure 1.

**Table 1:** Summary of testing details for each organisation enrolled in the scheme.

Organisation	Number of individuals invited to participate	Number of Participants (%)	Total number of LFTs per day							PCR	Number of individuals with a given total number of tests (0,1, 2, 3, …, or 7)							
			Day 1	Day 2	Day 3	Day 4	Day 5	Day 6	Day 7		0	1	2	3	4	5	6	7
Mersey Police	1965	1358 (69%)	392	673	887	1080	1202	1358	1310	1301	2	52	95	142	199	215	281	372
Merseyside Fire & Rescue	96	90 (94%)	40	45	57	67	72	83	82	84	2	4	11	6	12	10	11	34
Alder Hey	233	182 (78%)	40	71	102	129	153	161	164	169	4	11	16	26	31	32	26	37
Wings Care	12	12 (100%)	7	10	11	12	12	12	12	12	0	0	0	1	1	1	2	7
Carers	1	1 (100%)	0	0	0	0	0	1	1	1	0	0	1	0	0	0	0	0
Local Government LSSL	7	4 (57%)	4	4	4	4	4	4	4	4	0	0	0	0	0	0	0	4
LCC	7	7 (100%)	0	1	3	6	7	7	6	7	1	0	0	2	2	2	1	0
Autism Initiative	2	2 (100%)	1	1	2	2	2	2	2	2	0	0	0	0	0	5	0	1
Rodney House Residential Home	1	1 (100%)	0	1	1	1	1	1	1	1	0	0	0	0	0	0	1	0

Note 1: There were 57 missing PCRs for Merseyside Police, consisting of 3 who were removed mid-pilot due to missing LFT, 3 who withdrew as a household member tested positive, 4 who withdrew due to developing symptoms, 3 who decided to withdraw and self-isolate, 8 who were enrolled too close to the end of the scheme, and 36 who reported doing a PCR, but no result was received.

Note 2: There were 6 missing PCRs for Merseyside Fire and Rescue consisting of 3 who withdrew during the pilot, and 3 who reported doing a PCR but no result was received.

Note 3: There were 13 missing PCRs for Alder Hey consisting of 4 who withdrew due to a household member testing positive, 1 who stopped working for Alder Hey mid pilot, 1 who withdrew consent mid-pilot, 1 who developed symptoms and chose to isolate, 1 who was isolating for a different medical condition, 3 who were withdrew from the pilot due to non-compliance, and 2 who reported doing a PCR, but no result was received.

Note 4: No positive LFTs were observed for any individuals with missing PCR results.

**Figure 2:**
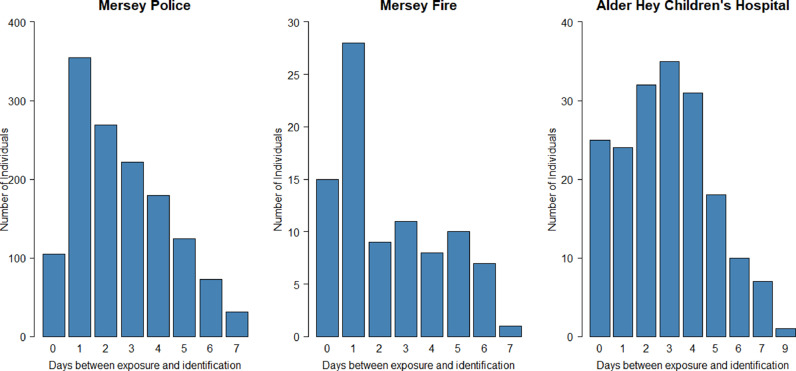
Number of days between exposure and identification of contact with a case.

Most individuals, 1358, enrolled from Merseyside Police, 90 were from Mersey Fire and Rescue and 182 from Alder Hey Children's Hospital. Other organisations contributed smaller numbers. The median number of days between exposure and identification as a contact of a case, was 2 days (interquartile range = (1, 3)). Figure 2 shows the distribution for the time between exposure and identification as a contact with a case for individuals from Mersey Police, Mersey Fire and Alder Hey. Most individuals in the Police and Fire services were identified within a few days of contact with an infected individual, whilst for Alder Hey identification occurred later in the week following contact with a case.

Over 70% of those eligible agreed to participate (Table 1). Conscious of the need to avoid over-burdening participating organisations, reasons for non-participation were not systematically recorded by organisations. However, based on feedback from organisational leads, most opt-outs from SMART-release were due to travel reasons. As the pilot only had limited LFT sites, some people (especially those who lived beyond Liverpool but were eligible participants due to working in the city) stated they did not want to commit to travelling to the test sites daily. The other main reason for not taking part was that many eligible participants had the necessary technology and means to work from home, negating the need to participate. Participation from smaller organisations was almost complete, but additional reasons from the larger organisations who saw higher numbers not participating included people preferring not to test so often, not trusting the results of the test, or being concerned about vulnerable family members.

Basic demographic features of participants from Merseyside Police, Merseyside Fire and Rescue and Alder Hey Children's Hospital are shown in Table 2. Most participants were white and aged between 30 and 50. We have not displayed information from organisations with fewer participants to maintain anonymity.

**Table 2:** Demographic features of individuals enrolled in the pilot from Police, Fire and NHS.

Organisation	Mersey Police	Merseyside Fire & Rescue Service	Alder Hey Children's Hospital
Number of Individuals	1358	90	182
Age (median, IQR)	36 (27, 46)	41 (32, 52)	35 (28, 45)
Males	899 (66·2%)	Not available	Not available
Females	459 (33·8%)	Not available	Not available
White	1315 (96·8%)	85 (94·5%)	157 (86·3%)
Non-White	43 (3·2%)	3 (3·3%)	11 (6%)
Not given	0	2 (2·2%)	14 (7·7%)

Note 1: Non-White ethnic groups have been merged to avoid identifiability.

Note 2: The sex of participants was not recorded by Merseyside Fire & Rescue Service or Alder Hey Children's Hospital.

Thirty-four individuals tested positive with PCR during the pilot, 31 from the Police, 1 from Merseyside Fire & Rescue Service, and 2 from Alder Hey Children's Hospital. Thirty-one of these individuals were identified first by LFT. The median number of days between an individual being identified as a contact and testing LFT positive was 1 day (interquartile range = (0, 3)).

**Figure 3:**
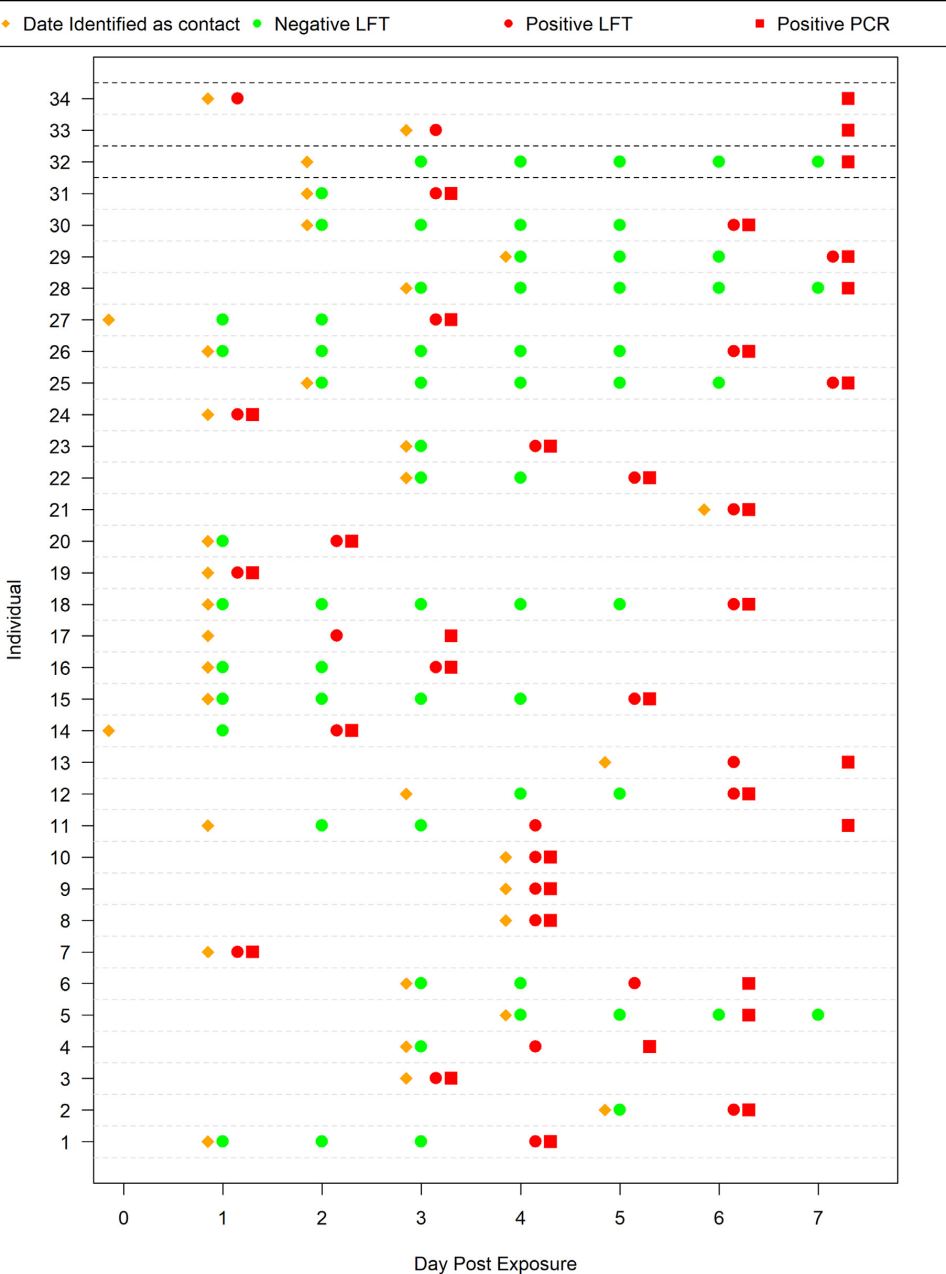
Graphical representation of the testing history of 34 individuals testing positive by PCR. Individuals 1-31 worked for Mersey Police, individual 32 worked for Mersey Fire, and individuals 33 and 34 worked for Alder Hey. Individuals 5, 28 and 32 were not identified by the daily LFT testing. Each circular point represents an LFT test (green=negative, red=positive). Red squares show a positive PCR, and orange diamonds show the date of identification as a contact of a known Covid-19 case.

Figure 3 shows a graphical representation of the time each of these 34 individuals were identified and their testing history. Table 3 summarises the accuracy of the serial testing scheme compared to evaluatory PCR results.

**Table 3:** Accuracy of serial LFT testing in Police workers. A positive LFT is defined as an individual who had at least one positive LFT at any point during their seven days of observation. The “No result” column denotes individuals with a missing PCR result for reasons described in the footnote to Table 1. 41 individuals with no result reported doing a PCR test but never received results whilst the remaining individuals withdrew from the pilot.

			PCR Result			
			void	Negative	Positive	No Result
Serial LFT	Police	All Negative	1	1269	2	57
		One Positive	0	0	29	0
	Mersey Fire	All Negative	0	83	1	6
		One Positive	0	0	0	0
	Alder Hey	All Negative	0	167	0	13
		One Positive	0	0	2	0

1588 individuals (participants who consistently tested negative and participated until day 7) were able to continue to work. A total of 8291 workdays would have been lost to self-isolation but were prevented due to negative daily tests.

### Key worker release scheme – police cohort

The total number of eligible participants in the police force was 1965, with 1358 participating in the scheme (69% participation rate).

The number of tests conducted by members of the police force identified as contacts is summarised in Supplementary Table S1. Compliance was high, with 6829 LFT tests conducted out of 7049 expected (96·9%). This is likely an underestimate, given that some individuals would have been notified late in the day that they were contacts, providing a short time window to being able to conduct an LFT test on the same day they were informed.

There were 57 individuals with a missing PCR result. Twenty-one of these withdrew from the study, for reasons described in Table 1. The remaining 36 individuals with a missing PCR reported having taken a PCR test but did not get a result from it. The reasons for this are unclear. None of these 36 individuals had a positive LFT result (Table 3).

There were 31 positive cases within the Police cohort shown in Figure 3. Merseyside Police had their own Test & Trace procedures in place which identified that one of these participants went on to infect 2 other participants. None of the other positive cases were linked.

Within the Police force SMART Release identified 29/31 cases (Sensitivity = 93·5%, 95% Confidence interval (78·6% to 99·2%)). This compares to a sensitivity across organisations of 31/34 = 91·2%, 95% Confidence interval (76·3% to 98·1%). No false positives were observed.

### Other organisations

Ninety workers from the Merseyside Fire and Rescue Service (MFRS) participated in this pilot. Three individuals withdrew from the study, and 3 individuals reported having taken a PCR test but did not get a result from it. One positive PCR was recorded, and this individual was not detected by the daily LFT testing (Table 3 and Individual 32 in Figure 3). Details of the number of tests relative to the day of identification are shown in Supplementary Table S2. Compliance with the daily testing scheme was 93·7% (446 tests out of an expected 476). As with Merseyside Police, this is likely to be an underestimate, since some individuals will have been notified too late in the day to do an LFT test on the day of notification, and will have started the following morning.

For Alder Hey Children's Hospital, 182 individuals entered the pilot. Eleven of these withdrew from the study, for reasons described in Table 1. The remaining two individuals with a missing PCR result reported having taken a PCR test but did not get a result from it. Two positive individuals were identified, and both returned positive LFTs and PCRs (Table 3 and Individuals 33 and 34 in Figure 3). Details of the number of tests relative to the day of identification are shown in Supplementary Table 3. Compliance with the daily testing scheme was 92·8% (805/867), again a likely underestimate.

For Wings Care, all twelve individuals have negative LFTs and a negative PCR.

Details of the remaining organisations can be found in Table 1. No positives were identified for these organisations either by LFT or by PCR. One individual at LSSL had a day 9 PCR instead of day 6/7 (7 days after being alerted rather than 7 days after exposure). Similarly, one of the Autism Initiative participants had a day 9 PCR and one of the carers had a day 6 and 7 LFT and then a day 8 PCR.

### Qualitative analysis

The following themes emerged following analysis of the extensive notes taken at the two meetings.

### Facilitators of engagement

The Implementation Pack was considered a useful guide, easily adapted to different organisational and sector needs.

Some organisations had their own internal systems such as in-house testing (Fire & Rescue), supplies of home LFT kits for staff (Alder Hey Children's Hospital), and own systems for contact tracing systems (The Police and Alder Hey). All of these made it easier for staff to engage in the pilot programme. As official testing venues become fewer in number as the pandemic progressed, other organisations began looking to set up in-house testing to prevent SMART Release from stalling due to lack of access to testing centres.

### Failing communications between local and national policies

Domiciliary care providers emphasised initial anxiety about whether the pilot by-passed the protocols in place for Test & Trace. They were re-assured that the pilot was approved by DHSC. The same sector reported that poor communication of national guidance and regulations had affected recruitment into this local pilot. The confusion was to do with who was considered a contact and whether indirect or secondary contacts (contacts of contacts) could return to work as normal. Staff isolating as a result of this confusion (i.e. staying off work because they had been in contact with another worker who had been identified as a contact of someone testing positive for Covid-19) had quite seriously impacted domiciliary care services.

It was felt that there were potential inconsistencies, and so muddled messages, between the SMART Release protocol and guidance issued by employers’ organisations. For example, the Care Quality Commission's Covid protocol publication aimed at care homes felt inconsistent with SMART Release.

A key benefit of SMART Release was that the protocol was clear and participation in the pilot scheme over-rode other, less clear communications, which domiciliary care staff felt overwhelmed by: *“too much guidance on everything”* and people *“don't have time to digest it all”*.

### Appropriate adjustments for sector needs

Following the issuing of implementation packs, initial conversations were held between local public health officials and the 35 adult social care providers who expressed an interest in taking part. These were felt to be helpful, providing detail and enabling discussion about specific organisational needs: *“there was no such thing as a daft question”*. These conversations were held remotely or there were dedicated drop in Q&A sessions and/or individual one-to-one meetings with managers/pilot coordinators.

Liverpool Street Scene Services Ltd (https://liverpool.gov.uk/business/liverpool-streetscene-services-ltd/) provided insights into how they had been able to flex their procedures to meet the needs of their workforce. Communications were adapted for this setting as many of the staff don't have access to a computer or smartphone. They reported having spread the word using leaflets in accessible language that were delivered within information boxes collected by refuse truck drivers daily. Their communications were sent out in the form of frequently asked questions.

The Fire Service's used electronic newsletters, and the Director of Public Health wrote letters for participants, raising awareness and credibility of the scheme.

### Critical mass

It became clear that how easy the SMART release pilot was to implement depended on the size and resources of the provider. Key factors included how big/small the organisation was; what capacity it had to get the message out efficiently and to support internal communications. For example, the police are well versed in running operations and have the infrastructure and ethos to support it. The LSSL SMART Release lead had good direct contact with managers and supervisors and so received daily updates about staff needing to isolate, meaning there was no lag in the system. By contrast, smaller providers in the domiciliary care sector had less capacity to manage implementation, communication, monitoring daily uptake of tests and reporting.

### Partnerships in pilot

Liverpool City Watch camera surveillance service reported that because of their close working partnership with the Police force, they understood the importance of SMART Release for maintaining a service. It was this close working relationship that led them to sign up to SMART Release. Harnessing the experiences of services who were further along in their involvement in the pilot proved a useful source of information for newer participant organisations with pilot programme coordinators supporting one another via the regular Steering Group meetings.

### Establishing a resilient system

The SMART release initiative was felt to be *“a really good lever”* for employers. Having it up and running in place and in a state of readiness to tackle subsequent phases of the pandemic helped to make service provision feel more secure, stable, and sustainable. However, some issues were highlighted that needed addressing to establish system resilience.

1. A need to improve the speed and level of detail of inputs into the reporting system. Systems for collecting and monitoring data must be in place early on, with sustainable capacity for supporting data collation identified and resourced.
2. The strict implementation of the 24-h gap requirement between tests was problematic for shift workers. The message could be more flexible, such as for staff to have a negative test before coming into work for their upcoming shift.
3. For some smaller organisations, the identification of eligible participants, getting them to join, gaining consent and enrolling could take as long as the normal isolating period.
4. Capacity issues mid-outbreak were a challenge for some. The Police, who were very well set up, struggled with admin capacity issues in the middle of the third wave, when hundreds of their staff were eligible. A need to build flexible capacity into the system to deal with capacity issues at peak was therefore identified.
5. Availability of community testing venues. The reduction in number of official community testing venues affected organisations, particularly domiciliary care providers as 41% of their workforce did not have access to a car. Organisations were particularly keen to introduce home testing. However, there were reservations about introducing unassisted testing in case this affected the reliability of lateral flow devices, particularly with the emergence of the Delta Variant. UK Health Security Agency launched an England-wide pilot in May 2021 which did consider self-testing at home as an alternative to self-isolation.

## Discussion

Daily contact testing (test-to-release) with lateral flow devices helped to sustain services under staffing pressures due to Covid-19. There were 34 SARS-CoV-2 infections identified and only 3 of these were missed by the daily testing regime among a total of 1581 participants (completing the regimen with an exit PCR). Only two individuals were known to be connected to subsequent onwards infections within an organisation, suggesting that most positive cases were still caught before they became infectious to colleagues.

If 16·3% of contacts of known Covid-19 cases test positive,^2^ and daily LFT testing identifies 31/34 individuals who test positive within 7 days of contact, this suggests that for every 100 contacts of known cases, daily LFT testing would identify approximately 14-15 of 16 individuals who go on to test positive. Clearly, several factors influence this approximate calculation, including the viral load of the original contact, the amount of mixing of individuals with other workers, and the changing prevalence and nature of SARS-CoV-2 variants over time. But daily testing in is likely to identify most Covid-19 cases, whilst allowing key services to still function. There remains a risk of transmission in the early stages of infection before a positive LFT result is available, but the overall risk-mitigation in this study was sufficient.

Previous concerns over the proportion of cases LFTs miss are addressed when considering their ease of deployment, rapid results and that these tests are more likely to pick up those individuals with more virus and thus more likely to be infectous.^4^ Since the individuals on the SMART Release scheme were likely to be at the start of their infection cycle (if infected at all), daily testing appears to have allowed them to be picked up early, as soon as their viral load starts to increase to levels detectable by LFT. There were no false positives observed in our study in 8322 lateral flow tests. This agrees with the findings of a large Canadian study of regular testing in the workplace which reports approximately 1 false positive in 4300 tests.^18^ In addition, the daily testing regimen over a period of 7 days means it is less likely cases will be missed.

From February 2021, participants could have a PCR test delivered to their home. It is possible that this change may have introduced a bias, if the swabbing quality was less good than self- tests. However, all the participants conducted their own PCR tests and were given the same instructions irrespective of whether the PCR test was taken at a test centre, or at home. This is likely to reduce the risk of bias. Even assisted tests are subject to less accuracy than clinician led tests.^19^ In addition, despite the risk of swabbing quality bias, there are reports of high confidence in the public's ability to perform tests correctly.^13^ Participants were able to access any community testing centre in Liverpool or at pop-up centres in Merseyside Police and Merseyside Fire & Rescue Service. This managed access to tests overcame problems Public Health England reported in their daily contact testing pilot using home testing.^13^

We are aware of one participant who tested negative initially and was linked to two subsequent cases before being identified. This was identified early thanks to effective monitoring of contacts by Merseyside Police and there were no further infections. Previous studies have shown that concerns about the accuracy of LFT tests were a barrier to the use of daily LFT instead of quarantine.^12^ However, our study suggests that repeat testing may overcome some of the concerns over the accuracy of single LFTs.

There were many changes in the prevalence of Covid-19, and subsequently the local measures that were implemented in Merseyside during the SMART Release pilot. There was a peak in cases in January 2021 followed by a national lockdown restricting mixing outside work and so reducing transmission routes. England has since gone through a “roadmap” out of restrictions, now with very little social distancing, requirement to wear face coverings or certification of vaccination or a recent negative test result for access to indoor social spaces. This situation may change as the Omicron variant becomes ubiquitous. Daily contact testing regimens need to adapt promptly to changing prevalence, viral characteristics, and restrictions.

As the pandemic progressed in the UK, from 14th December 2021, individuals who had been fully vaccinated and were contacts of a known Covid-19 case were able to use daily testing for 7 days rather than isolation. Our study contributes to understanding the impact of such a policy. Along with a Public Health England survey,^12^^,^^13^ it suggests that compliance with a daily testing regime as an alternative to isolation is likely to be high. Our study also adds that most infections are likely to be detected through daily testing with LFT. However, no conclusions can be drawn about how increased exposure prior to detection with LFT leads to more cases than complete isolation would, since we didn't have complete onwards transmission data.

Our paper provides portable evidence on daily testing as an alternative to quarantine. Although Covid-19 restrictions have been largely lifted in the UK at the time of writing, restrictions may remain in high-consequence settings or be reintroduced in the event of new variants of SARS-COV-2 or similar threats in the future. An analysis of the costs of serial testing schemes would be useful to determine the optimal frequency of serial testing, relative to the prevalence/risk of infectious disease.

Over 70% of eligible participants preferred to take part in the study rather than quarantine. This is consistent with findings of one other study considering daily testing in the general population.^13^ Engagement with the SMART-Release scheme was good, with over 95% adherence. This contrasts with findings on the use of LFTs in care homes.^16^ The locally supported implementation pack, adaptive protocol and clear messaging were key to the scheme's success at a time when services experienced “too much guidance” and were feeling overwhelmed. The project team and workplace coordinators collaborated well, adapting to organisational needs, holding drop in question-and-answer sessions, and one-to-one meetings as needs arose. The steering group, expert guidance and connections with the Covid-SMART pilot between national and local agencies worked well. The steering group meetings with researchers were not recorded. This raises the possibility of recall bias in the qualitative aspects of this study. However, given that detailed notes were compiled by two researchers, who then compiled their findings before performing the thematic analysis, we believe this risk is minimal.

There were limitations: We do not have detailed data on reasons for not participating in the pilot. However, feedback from the organisations highlighted key factors being ease of working from home, and difficulty of access to testing centres, both of which were also identified in a Public Health England Study.^12^

We lacked cycle threshold values for inferring viral loads because of the PCR platform used. Having this data may have allowed us to infer reasons for the negative LFT tests where positive cases were missed by the daily testing regime. For example, if cycle threshold values were high (indicating low viral load), LFT would not be expected to identify these cases, likely to represent individuals at the very beginning or the end of their infection cycle. There were 41 missing PCR results from those who reported going for their evaluation PCR test but who did not receive their results. Each organisation confirmed that the individuals in question reported going for their test and that the results were not sent. It is not known whether this is due to issues with lab processing or result dataflows at a time when NHS Test & Trace was processing tests at an unprecedented scale. It is reasonable to assume that almost all these missing results were negative since none of the individuals concerned subsequently reported symptoms or positive tests. Additionally, all these individuals returned only negative results in their daily LFT testing and none of them reported being contacted by NHS Test & Trace as would be expected if they had tested positive. As a sensitivity analysis, if we assume that the prevalence of positives in these missing 41 tests was similar to that in the individuals with known PCR results (34/1554), then there is approximately a 94% chance of observing 2 or fewer positives in these missing results. If one of the missing PCRs was actually positive, the reported sensitivity would be 88·6% (95% CI: 73·3%, 96·8%), and if two were positive the sensitivity would be 86·1% (95%CI 70·5%, 95·3%). However, as stated, we think it is most likely that these missing PCR results were negative results.

There was anxiety about whether this local pilot bypassed national protocols from NHS Test & Trace, despite it being approved by DHSC. Participation was hindered by several factors, including reluctance to travel for those that lived far from testing sites. The pilot was resource intensive and easier for organisations with additional resources, such as Merseyside Police, to run.

The protocol required photographic evidence of test results to be sent to an individual's pilot co-ordinator. This did not happen on all occasions. An online survey of participants was planned upon completion of the pilot to learn lessons for future implementations of similar schemes. However, due to capacity restraints, this part of the pilot did not happen and focus groups were relied on to qualitatively assess the pilot.

Adherence to SMART Release was good. The results of this pilot suggest that daily testing of key workers can maintain services without posing serious risk to the wider workforce. With effective monitoring and oversight, 1588 individuals (participants who consistently tested negative and participated until day 7) were able to continue to work. A total of 8291 workdays would have been lost to self-isolation but were prevented due to negative daily tests. All but one individual with Covid-19 were identified before they subsequently infected others, safeguarding critical services from what could otherwise have been severe disruption.

Daily testing provides a useful alternative to quarantine for contacts of SARS-CoV-2 infected individuals, which is particularly valuable for sustaining essential services, and thus public safety. Key enablers were clear communication over a meaningful protocol, and flexible support for workplaces from the local public health team. Enabling participants and workplaces to learn from each other's experiences, in changing circumstances, is likely to be important to the ongoing success of daily contact testing.

## Funding

This report is independent research commissioned by DHSC and part funded by DHSC and NIHR. Further funding was received from Liverpool City Council, the EPSRC and MRC.

## Contributors

L.M., E.C. and M.A. are part of the Liverpool City Council Public Health Department and managed the delivery of the project within the overall Covid-SMART programme with IB, MA and EC on Gold Command. D.M.H. and C.C. managed the data and carried out analysis. L.M. and D.M.H. drafted the initial manuscript under the supervision of M.G.F., I.B. and R.C. The corresponding author attests that all listed authors meet authorship criteria and that no others meeting the criteria have been omitted.

## Data sharing statement

Pseudonymised data are accessible via CIPHA. Requests can be made to the Data Asset and Access Group for extracts of the larger-scale data which cannot be released openly due to information governance requirements. All R code is accessible from the corresponding author.

## Declaration of interests

IB reports consultancy fees from Astra Zeneca for work not related to this manuscript, and is a member of the UK Testing Initiatives Evaluation Board. IB received grants from the Department of Health and Social Care and from the National Institute for Health Research. DMH reports a small grant from the Psoriasis and Psoriatic Arthritis Alliance for work unrelated to this manuscript. DMH received grants from the Department for Health and Social Care, MRC and Halton Borough Council. MGF reports participation on advisory boards for UK DPFS, UK NC3Rs, and HRCI-HRC-JFS-2022 - Review Panel. This was unrelated to this work. MGF received grants from the Department for Health and Social Care and Halton Borough Council. All the other authors report no conflicts.

## Appendix Supplementary materials

Image, application 1

Image, application 2
