# Supplementary material for: Daily testing of contacts of SARS-CoV-2 infected cases as an alternative to quarantine for key workers in Liverpool: A prospective cohort study
Source: eClinicalMedicine. 2022 Jul 1;50:101519. doi: 10.1016/j.eclinm.2022.101519 (PMC9249302; doi:10.1016/j.eclinm.2022.101519)
Supplement: Supplementary file 1 [file mmc1.docx]

# **SMART Release Template Protocol v1.0**

## **Background**

The SMART Release pilot for key workers will enable staff to safely reduce avoidable quarantine. This is a nationally authorised programme.

Currently, individuals who have been in contact with a confirmed case of COVID-19 need to quarantine for 10 days since their last exposure. Liverpool City Council is working in partnership with key agencies in Liverpool on a SMART-Release pilot for key workers.

Individual staff who will be eligible to participate in the pilot are:

- close contacts of confirmed cases of COVID­19 (outside their own household/bubble)
- contacts must live or work in Liverpool

Under the SMART Release pilot staff contacts must commit to have serial daily tests with Lateral Flow Tests (LFTs) up to day 7 after their exposure with a confirmed case. If they test negative, they are released from quarantine for 24 hours until their next test. On day 6 or day 7 post exposure, the staff member has both an LFT and a PCR test (for evaluation).

## **Eligibility**

In order to participate in the Test to Release program the following criteria must be met:

- Individual must live or work in Liverpool
- Individual must have been identified as a close contact of a confirmed positive Covid-19 case either by NHS Test & Trace (App or caller) or otherwise
- Individual must commit to undertake serial daily testing commencing from day 1 post exposure until Day 7 following individual’s last contact with a positive case
- Each daily rapid Lateral Flow Test must return a negative result, and individual is then released from quarantine for 24 hours.
- Participation in the pilot is voluntary.
- Individual is not eligible if there is a confirmed COVID case in their household or bubble, or if anyone in their household or bubble has symptoms of coronavirus and therefore should be tested.

If the individual meets the criteria and commit to the full period of rapid testing then under the Test to Release protocol, the individual can be released from quarantine on a daily basis either to attend work or to carry out routine activity permitted in accordance with the localised rules (lockdown or tier restrictions applicable to where they live.)

Close contacts who are not able to commit to the full period of daily testing must continue to quarantine in accordance with Government guidance & NHS Test & Trace Guidance.

## **General Guidance**

1. Under the protocol staff identified as contacts (non-household/bubble) must undertake rapid LFT each day up to and including day 7 after the last day of contact with the positive case. The day of contact/exposure is day zero.

Staff should ensure that there is no more than 24 hours between their tests.

- - Please insert details here about setting specific arrangements, e.g. whether or not individuals can have a test in their usual workplace (such as within Care Homes where this is already set up) or whether staff should access the lateral flow test at any of the community testing sites as usual

1. If individual gets a negative LFT result, they can be released from quarantine for a period of 24 hours until their next test. They must still follow all COVID rules:
   - stay 2 metres apart from people they do not live with.
   - wear a face covering in public indoor settings where social distancing may be difficult, and where they will come into contact with people they do not normally meet, including outdoors.
   - wash their hands often and as soon as they get home, wash their hands regularly and for at least 20 seconds.
2. Each day, participants must send a photo or screenshot of their test result to your pilot coordinator (add in details here).
3. On day 6 or 7 after the individual was last exposed to the confirmed case, in addition to their daily Lateral Flow Test they need to book a PCR test online to help us evaluate the pilot.
   - The lateral flow test can be accessed at any of the community testing sites or their organisation testing site as usual AND
   - The individual must book a PCR test at a separate walk-through or drive-through test site at <https://www.gov.uk/get-coronavirus-test>
   - When the individual participant is booking the PCR test, they should select “yes” when asked if they are taking part in a government pilot project. When asked which trial it is, select *‘community testing’.*
   - Same day appointments may depend on availability, so participants should check online in advance to ensure they can book an appointment for their 6^th^ or 7^th^ day.
   - If an individual would prefer a home test to be posted to their home, they should book this by 3pm on day 5 to make sure the kit arrives in time to do the test on day 6 or day 7 post exposure.
4. There is no requirement to quarantine whilst awaiting the results of this PCR test. Participants are released from quarantine if the final LFT on day 7 post exposure is negative. Individual participants must send the result of the PCR test to their organisation’s pilot coordinator as with all their test results on a daily basis.
5. In the unlikely event that the PCR test done on day 6 or day 7 is positive despite serial negative LFTs, an individual assessment will be made by Director of Public Health as to whether any public health action is required.
6. If at any stage, a participant develops symptoms, they and anyone they live with or who is part of their social bubble must self-isolate, until they get a PCR test-result. It is not sufficient to rely on a negative LFT result if they have symptoms. They must inform (insert details of the organisation’s pilot coordinator). The main symptoms of coronavirus are:

- a high temperature
- new, continuous cough
- loss or change to sense of smell or taste

## **Positive tests**

- If a participant gets a positive LFT result, they and all members of their household/bubble need to isolate immediately for 10 days.
- In order to confirm that the result is positive, the individual must book a PCR test at Hunter Street testing site via <https://www.liverpoolccg.nhs.uk/confirmatory-pcr-test> or call 0845 111 0692. When booking they should select that they require ‘Confirmatory PCR Testing’.
- If confirmatory PCR test confirms positive result, continue to isolate in line with Government Guidance from date of initial positive LFT.
- If confirmatory PCR test returns a negative result (following positive LFT test) then the pilot can continue and daily LFT tests recommence up to and including Day 7 post exposure.

## **Example scenario**

- You are notified on Tuesday 5^th^ January that you are a contact of positive case and your last exposure was on Saturday 2^nd^ January.
- Day 0 is therefore Saturday 2^nd^ January (date of last contact with positive case) and Tuesday 5^th^ is Day 3
- LFT undertaken on Day 3 and if negative you are released from quarantine for 24 hour period until your next test.
- LFT repeated on Days 4, 5, 6, 7 all returned negative
- PCR test at end of testing regimen undertaken on Day 6 or 7 booked via <https://www.gov.uk/get-coronavirus-test>.
- You are not required to quarantine whilst awaiting the final PCR result

## **Monitoring and Evaluation**

All details of daily testing must be submitted to the organisation’s Pilot Coordinator:

- Name
- Contact email and phone number

This should include a photo/screenshot of tests results if testing is not done in the workplace.

Each participating organisation must share anonymised report with Liverpool City Council and the University of Liverpool on a weekly basis by 5pm every Monday.

Participants should be asked for their consent to share their data as set out in the individual participant letter in the implementation pack. This also includes consent for data linkage to be made with other COVID test databases. Organisations are responsible to ensure that they have received and retain written consent from all participants.

Participants will be required to complete a short online survey to enable us to learn lessons and inform future plans. This will be sent to organisations by the project support team for the organisation to send to participants at the end of their testing regimen and to follow up to ensure high return.

We will also gather feedback from organisations about lessons learned and case studies and may hold focus groups. Case-studies we would like to include in the evaluation report will be discussed with participating organisations beforehand to make sure they approve.
