# Supplementary material for: Daily testing of contacts of SARS-CoV-2 infected cases as an alternative to quarantine for key workers in Liverpool: A prospective cohort study
Source: eClinicalMedicine. 2022 Jul 1;50:101519. doi: 10.1016/j.eclinm.2022.101519 (PMC9249302; doi:10.1016/j.eclinm.2022.101519)
Supplement: Supplementary file 2 [file mmc2.docx]

**Supplement to: Daily testing of contacts of SARS-CoV-2 infected cases as an alternative to quarantine for key workers in Liverpool: a prospective cohort study.**

# **Authors**

Lucy Marsden^*^, Speciality Registrar in Public Health^1^, David M Hughes^*^, Lecturer in Health Data Science^2^, Prof. Rhiannon Corcoran, Professor of Psychology & Public Mental Health^3^, Christopher P Cheyne, Postdoctoral Research Associate^2^, Matt Ashton, Director of Public Health^1^, Iain Buchan^**^, Executive Dean^4^, Emer Coffey^**^, Consultant in Public Health^1^, Marta García-Fiñana^**^, Professor of Health Data Science^2^

^*^ joint first authors; ^**^ joint senior authors

# **Affiliations**

*^1^* *Public Health Department, Liverpool City Council, Liverpool, Cunard Building, Water Street, Liverpool, L3 1DS*

^2^ *Department of Health Data Science, Institute of Population Health, University of Liverpool, Liverpool, UK*

^3^ *Department of Primary Care and Mental Health, Institute of Population Health, University of Liverpool*

^4^ *Department of Public Health, Policy and Systems, Institute of Population Health, University of Liverpool, Liverpool, UK*

**Details of the SMART release pilot**

For organisations to join the pilot, a small project team led by the local Public Health Team (Liverpool City Council) developed an implementation pack comprising key documents including briefing notes, a template protocol with guidance, a monitoring spreadsheet and consent statements. These documents ensured rapid implementation was possible, with organisations equipped for briefing staff, inviting them to participate and to collate the necessary data. A steering group was established to oversee and support the extension of the pilot.

PCR testing initially took place at a pillar 1 testing centre (Hunter Street, Liverpool) and from February 2021, participants could book a PCR test via the gov.uk website which would be delivered to their home address.

Managers from each participating organisation were routinely informed if a member of their staff was identified as a contact of a positive case. This enabled them to invite the individual to take part in the scheme.

The scheme was run until the submission of the full Liverpool Covid-SMART Community Testing Pilot report. At this time, national daily contact testing (DCT) studies led by DHSC and Public Health England (PHE) were running. In June 2021, the scheme came to an end for the organisations with smaller numbers of participants, including Liverpool Street Scene Limited and Domiciliary Care providers. For organisations where staff were deemed to be working in critical roles and where there were significant concerns for workforce capacity due to rising Covid-19 prevalence, the scheme continued until 16^th^ August 2021 (Alder Hey Children’s Hospital, Merseyside Police and Merseyside Fire & Rescue Service).

Organisations would monitor the daily test results and outcome of the confirmatory PCR using the monitoring spreadsheet included within their implementation pack. This was anonymised by each organisation on a weekly basis and shared with the Project Team (LCC) who reviewed it for accuracy and to check there were no gaps in the data. Any missing data was queried with the organisation who checked their reporting and added any missing fields. The data was then shared with University of Liverpool who carried out an independent quantitative analysis.

*Table S1: Number of individuals in Mersey Police identified as contacts on the day of the exposure and 1, 2, 3, 4, 5, 6 and 7 days post exposure. The number of expected LFT tests, as well as the number of LFTs and PCR tests conducted are shown. The number of tests expected corresponds to the number of days from the day the individual is identified as a contact till Day 7 (or till withdrawal). The cells highlighted in blue captures individuals with complete follow-up from the day they were identified as contacts, and it takes into account that some individuals may have been notified late in the day and it is the next day when they can start testing. Withdrawals are denoted w, the end of the pilot e and positive cases^+^.*

| Day identified as contact | Number of individuals | Total number of LFT tests taken by an individual. | | | | | | | | | LFTs reported | LFTs expected from day of notification | | PCRs reported | | Missing PCRs | |
| --- | --- | --- | --- | --- | --- | --- | --- | --- | --- | --- | --- | --- | --- | --- | --- | --- | --- |
|  |  | 0 | 1 | 2 | 3 | 4 | 5 | 6 | 7 |  | |  |  | |  | |  |
| 0 | 105 | 0 | 0 | 1^+^, 2^e^ | 1^+^ | 0 | 0 | 0 | 101 | 716 | | 716 | 101 | | 4 | |  |
| 1 | 355 | 0 | 4^+^, 2^w^ | 1^+^, 1^e^ | 2^+^ | 1^+^, 5^e^ | 1^+^ | 69, 2^+^ | 267 | 2340 | | 2409 | 338 | | 17 | |  |
| 2 | 269 | 0 | 1^w^ | 1^+^ | 0 | 0 | 53, 1^+^ | 212, 1^+^ |  | 1557 | | 1610 | 258 | | 11 | |  |
| 3 | 222 | 1^w^ | 1^+^, 2^w^ | 2^+^ | 3^+^, 1^w^ | 51 | 160, 1^+^ |  |  | 1028 | | 1079 | 212 | | 10 | |  |
| 4 | 179 | 0 | 3^+^, 3^w^ | 1^w^ | 30 | 140, 2^+^ |  |  |  | 666 | | 696 | 174 | | 5 | |  |
| 5 | 124 | 1^w^ | 1^+^ | 16, 1^+^ | 105 |  |  |  |  | 350 | | 366 | 121 | | 3 | |  |
| 6 | 73 | 0 | 1, 1^+^, 1^w^ | 68, 1^+^ |  |  |  |  |  | 141 | | 142 | 70 | | 3 | |  |
| 7 | 31 | 0 | 30, 1^+^ |  |  |  |  |  |  | 31 | | 31 | 27 | | 4 | |  |
| Total: | 1358 | 2 | 52 | 95 | 142 | 199 | 161 | 284 | 368 | 6829 | | 7049 | 1301 | | 57 | |  |

Table S2: Number of individuals in Mersey Fire identified as contacts on the day of the exposure and 1, 2, 3, 4, 5, 6 and 7 days post exposure. The number of expected LFT tests, as well as the number of LFTs and PCR tests conducted are shown. The number of tests expected corresponds to the number of days from the day the individual is identified as a contact till Day 7 (or until withdrawal). The cells highlighted in blue captures individuals with complete follow-up from the day they were identified as contacts, and it takes into account that some individuals may have been notified late in the day and it is the next day when they can start testing. Withdrawals are denoted ^w^, the end of the pilot ^e^ and positive cases ^+^.

| Day identified as contact | Number of individuals | Total number of LFT tests taken by an individual. | | | | | | | | | LFTs reported | LFTs expected from Day of notification | | PCRs reported | | Missing PCRs | |
| --- | --- | --- | --- | --- | --- | --- | --- | --- | --- | --- | --- | --- | --- | --- | --- | --- | --- |
|  |  | 0 | 1 | 2 | 3 | 4 | 5 | 6 | 7 |  | |  |  | |  | |  |
| 0 | 15 | 1^w^ | 0 | 0 | 0 | 0 | 0 | 4 | 10 | 94 | | 98 | 14 | | 1 | |  |
| 1 | 29 | 0 | 0 | 0 | 1^w^ | 0 | 0 | 4 | 24 | 195 | | 199 | 28 | | 1 | |  |
| 2 | 9 | 1^w^ | 0 | 0 | 0 | 0 | 4, 1^+^ | 3 |  | 43 | | 47 | 7 | | 2 | |  |
| 3 | 11 | 0 | 0 | 0 | 1 | 5 | 5 |  |  | 48 | | 55 | 11 | | 0 | |  |
| 4 | 8 | 0 | 0 | 0 | 1 | 7 |  |  |  | 31 | | 32 | 7 | | 1 | |  |
| 5 | 10 | 0 | 0 | 7 | 3 |  |  |  |  | 23 | | 30 | 9 | | 1 | |  |
| 6 | 7 | 0 | 3 | 4 |  |  |  |  |  | 11 | | 14 | 7 | | 0 | |  |
| 7 | 1 | 0 | 1 |  |  |  |  |  |  | 1 | | 1 | 1 | | 0 | |  |
| Total: | 90 | 2 | 4 | 11 | 6 | 12 | 10 | 11 | 34 | 446 | | 476 | 84 | | 6 | |  |

Table S3: Number of individuals in Alder Hey identified as contacts on the day of the exposure and 1, 2, 3, 4, 5, 6 and 7 days post exposure. The number of expected LFT tests, as well as the number of LFTs and PCR tests conducted are shown. The number of tests expected corresponds to the number of days from the day the individual is identified as a contact till Day 7 (or till withdrawal). The cells highlighted in blue captures individuals with complete follow-up from the day they were identified as contacts, and it takes into account that that some individuals may have been notified late in the day and it is the next day when they can start testing. Withdrawals are denoted w, the end of the pilot ^e^ and positive cases ^+^.

| Day identified as contact | Number of individuals | Total number of LFT tests taken by an individual. | | | | | | | | | LFTs reported | LFTs expected from Day of notifcation | | PCRs reported | | Missing PCRs | |
| --- | --- | --- | --- | --- | --- | --- | --- | --- | --- | --- | --- | --- | --- | --- | --- | --- | --- |
|  |  | 0 | 1 | 2 | 3 | 4 | 5 | 6 | 7 |  | |  |  | |  | |  |
| 0 | 25 | 0 | 0 | 0 | 0 | 1^w^ | 0 | 1 | 23 | 171 | | 172 | 24 | | 1 | |  |
| 1 | 24 | 0 | 1^+^ | 1, 1^w^ | 0 | 0 | 1, 1^w^ | 7 | 12 | 141 | | 149 | 22 | | 2 | |  |
| 2 | 32 | 0 | 1^w^ | 0 | 1^w^ | 1 | 11 | 18 |  | 171 | | 184 | 29 | | 3 | |  |
| 3 | 35 | 1^+^, 1^w^ | 0 | 1^w^ | 2^w^ | 11 | 19 |  |  | 147 | | 158 | 31 | | 4 | |  |
| 4 | 31 | 1^w^ | 0 | 2, 1^w^ | 8 | 18 |  |  | 1 | 102 | | 114 | 28 | | 3 | |  |
| 5 | 18 | 0 | 1 | 3 | 14 |  |  |  |  | 49 | | 54 | 18 | | 0 | |  |
| 6 | 10 | 0 | 2 | 7 | 1 |  |  |  |  | 19 | | 30 | 10 | | 0 | |  |
| 7 | 7 | 1 | 5 |  |  |  |  |  | 1 | 5 | | 6 | 7 | | 0 | |  |
| Total: | 182 | 4 | 10 | 16 | 26 | 31 | 32 | 26 | 35 | 805 | | 867 | 169 | | 13 | |  |
| Note 1 individual at Alder Hey was identified on day 9 after contact so not eligible for scheme.  One individual was identified on Day 7 and began their 7 LFTs at that point with PCR on day 14. One individual was identified on day 4 and began their 7 days at that point. These are noted in the table but not considered in the calculations of tests done and expected number of tests. They have been included in the counts of number of individuals and the counts of PCRs. | | | | | | | | | | | | | | | | |  |
